# Effects of electroacupuncture on cognitive symptoms in major depressive disorder: a pilot study and randomized controlled trial

**DOI:** 10.12688/f1000research.146897.4

**Published:** 2024-12-23

**Authors:** Yindee Boontra, Chommakorn Thanetnit, Muthita Phanasathit

**Affiliations:** 1 Department of Psychiatry, Thammasat University, Pathum Thani, 12120, Thailand; 2 Center of Excellence in Applied Epidemiology, Faculty of Medicine, Thammasat University, Bangkok, Bangkok, 12120, Thailand

**Keywords:** Electroacupuncture, Depression, Cognition, Humans, Pilot, Randomized Controlled Trial

## Abstract

**Objectives:**

To investigate the impact of electroacupuncture on cognitive function, quality of life (QoL), and depression severity in patients with major depressive disorder (MDD).

**Methods:**

This double-blinded randomized controlled trial included 60 participants aged 18-55 with cognitive symptoms related to MDD at Thammasat University Hospital. Participants were divided into two groups: the electroacupuncture group combined with standard antidepressant treatment (EG; n=30) and the control group receiving standard care with placebo acupuncture (CG; n=30). The study assessed 1) executive functions using the Trail making test- B and Stroop Color and Word Test, 2) delayed recall, and 3) subjective cognitive complaint and Quality of life (QoL) using WHODAS 2.0. Depressive symptoms were measured using the Thai version of the Patient Health Questionnaire (PHQ-9). Baseline and post-intervention assessments were conducted over 10 weeks. Mann-Whitney U test analyzed treatment effects by comparing median differences between groups.

**Results:**

Both groups exhibited similar demographics and cognitive traits. Cognitive improvement was observed in both groups at the endpoint. Intention-to-treat analysis revealed significantly higher median scores for subjective cognitive complaints in the EG compared to the CG (EG: Median = 5.5, CG: Median = 0.0, p=0.049). No serious side effects were identified from either electroacupuncture or placebo acupuncture.

**Conclusions:**

Electroacupuncture improved subjective complaints in MDD patients with cognitive symptoms, but did not show effects on specific cognitive functions, QoL, or depressive symptoms. This study provides initial evidence supporting the potential of electroacupuncture in MDD patients with cognitive symptoms, suggesting opportunities for further research.

**Trial registration:**

NCT06239740, February 2, 2024, ClinicalTrials.gov.

## Introduction

Depressive disorder is currently considered a significant health issue, with depression affecting up to 264 million people worldwide. Depression has both short-term and long-term consequences, with the most severe impact being suicide. Approximately 800,000 people die by suicide each year, making it the second leading cause of death among individuals aged 15-29 years. According to the World Health Organization (WHO). Approximately 1.5 million people in Thailand suffer from depressive symptoms. ^1^

Depression leads to cognitive dysfunction, affecting executive function and working memory. ^2^^,^^3^ This results in decreased attention and slow thinking, which are key diagnostic criteria for depression according to the Diagnostic and Statistical Manual of Mental Disorders (DSM-5). Even after depressive symptoms have improved, cognitive problems may persist, particularly in terms of work performance. ^3^

Current research indicates that various treatments may help alleviate cognitive dysfunction in patients with depression. Medications like duloxetine have been shown to improve psychomotor cognitive function, ^4^ while vortioxetine has demonstrated benefits in executive function, attention, processing speed, and learning and memory. Additionally, treatments such as Electroconvulsive therapy (ECT) and repetitive transcranial magnetic stimulation (rTMS) have shown potential in improving cognitive function in depressive patients. ^5^^–^^7^

Acupuncture, a traditional eastern medicine practice, has been used for over 4,000 years in China. The practice involves inserting tiny needles into specific points on the body to restore balance and harmony. Acupuncture has gained popularity due to its minimal side effects. Some studies have suggested that acupuncture may aid in the treatment of cognitive impairment in patients with schizophrenia, dementia, or mild cognitive impairment. ^8^^–^^10^ It has also been found to improve cognitive function and reduce depressive symptoms. ^11^ Acupuncture may stimulate the release of substances like brain-derived neurotrophic factor (BDNF), ^9^ which is crucial for neural plasticity, supporting the growth of new neurons in the brain.

Electroacupuncture, defined as acupuncture with electrical stimulation, includes inserting needles at acupuncture points and applying a low-level electrical current through them. A small amount of electricity flows through the electrode, resulting in a gentle vibration or soft hum during the treatment, enhancing the effects of acupuncture. This shown to increase the release of vascular endothelial growth factor (VEGF) ^12^ and enhance cerebral blood flow and volume, ultimately improving motor function in ischemic stroke patients. ^13^ In animal studies, electroacupuncture has been found to stimulate the release of nitric oxide (NO) and reduce angiotensin II, leading to better blood vessel dilation in the brain. ^14^^,^^15^ The authors hypothesized that electroacupuncture has the potential to treat subjective cognitive complaints in depressive patients, which is a relatively new and promising area of research. It is a minimally invasive and well-tolerated treatment option.

## Methods

This trial was registered with the Human Research Ethics Committee of Thammasat University (Medicine), which approved the study (approval code: MTU-EC-PS-1-304/64) for the period from December 24, 2021, to December 23, 2022. The study adhered fully to international ethical guidelines, including the Declaration of Helsinki, The Belmont Report, CIOMS Guidelines, and the International Conference on Harmonisation-Good Clinical Practice (ICH-GCP). The research protocol detailed a double-blinded, randomized controlled trial (RCT) and pilot study conducted at a single center. The study aimed to evaluate the impact of electroacupuncture on specific cognitive functions in individuals with major depressive disorder (MDD) and cognitive dysfunction, compared to a control group. Both the experimental and control groups received standard pharmacological treatment for depressive disorders. This study was conducted during the Coronavirus Disease (COVID-19) pandemic, necessitating modifications due to safety concerns and social distancing measures. As a result, the study was adapted to a pilot design with 30 participants in each group. The preliminary findings are intended to guide and inform future large-scale research on the effects of electroacupuncture on cognitive dysfunction in patients with MDD. To enhance research transparency and public accessibility, the authors registered the study with the Thai Clinical Trials Registry ( www.clinicaltrials.in.th), under the identifier NCT06239740, certified on February 2, 2024.

A total of 60 eligible participants, aged between 18 and 55 years, with cognitive symptoms resulting from MDD, were recruited from the psychiatry outpatient clinic at Thammasat University Hospital. All participants with MDD and low suicidal risk, diagnosed according to the DSM-5 ^16^ or DSM-IV-TR criteria, ^17^ and currently receiving standard treatment for at least 3 months from psychiatrists and psychiatric residents, were included. The subjective cognitive symptoms were screened using item 7 of the Thai version of the Patient Health Questionnaire (Thai-PHQ-9), ^18^ which assesses difficulties with concentration or cognitive problems. The question in item 7 reads, “Trouble concentrating on things, such as reading the newspaper or watching television).” The exclusion criteria were: (i) Individuals with severe cognitive deficits from traumatic brain injury, delirium, neurodevelopmental disorders, or intellectual disability; (ii) Individuals with neurological disorders such as stroke, Parkinson’s disease, epilepsy, or other brain lesions; (iii) Individuals with severe medical conditions preventing lying down for 20 minutes; (iv) Individuals with recent electroconvulsive therapy (ECT) within the last 6 months; (v) Individuals with a pacemaker; (vi) Individuals with visual or hearing impairment that could not be corrected with eyeglasses or hearing aids; and (vii) Individuals diagnosed with severe MDD or scoring 20 points or more on the Thai-PHQ-9. Each participant was provided with detailed information regarding the study and informed written consent was obtained for their participation in the research. Subsequently, all participants granted consent for publication.

**Figure 1.**
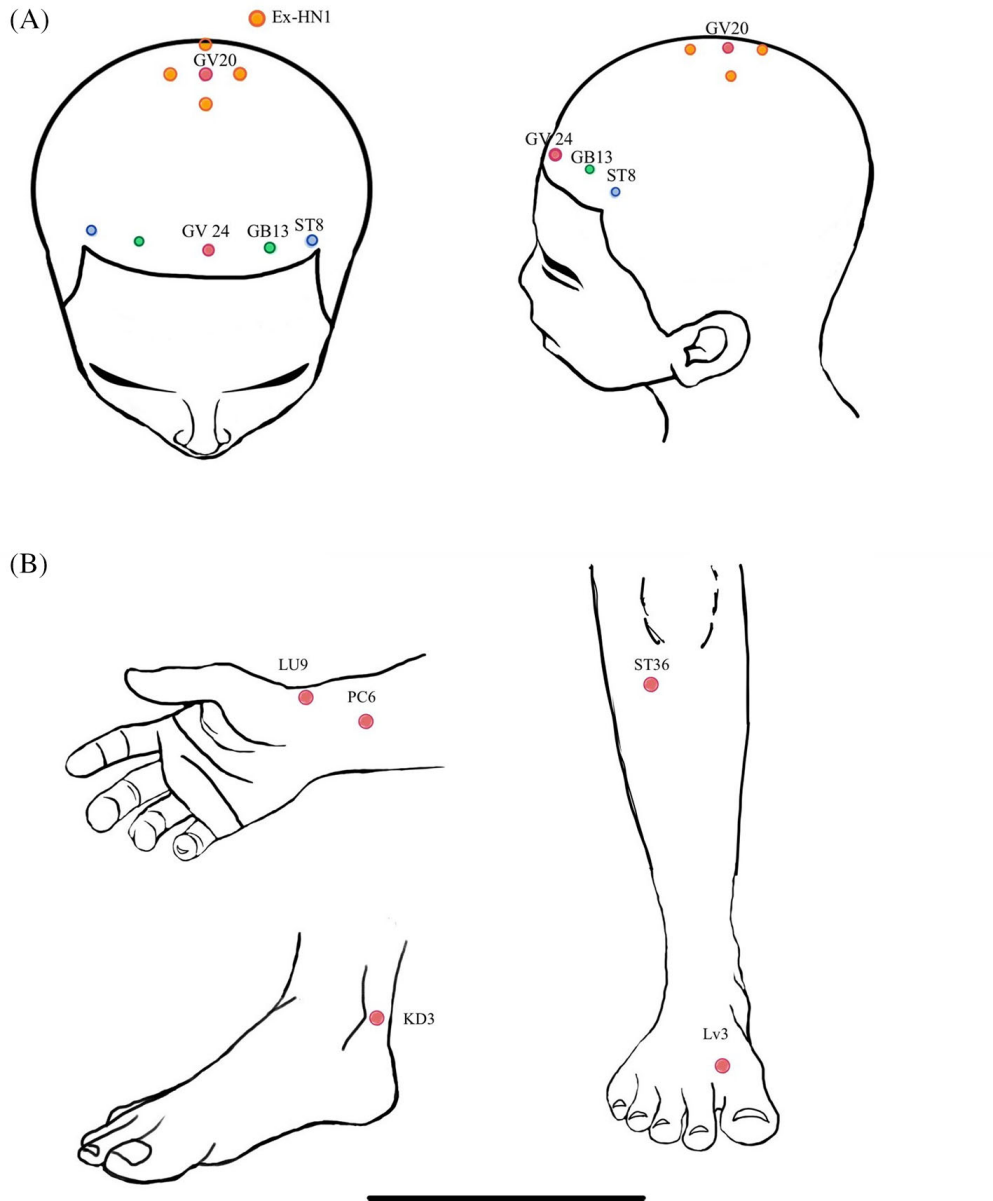
A) acupoint location at scalp region; B) acupoint location at extremities.

An assistant researcher, who had contact with participants but was not involved in the interventions and outcome assessment, assigned participants to the intervention or control arm using a block of four randomization. The participants were randomly assigned to either the treatment group or the control group in a 1:1 ratio. The treatment group was undergo electroacupuncture (EG), while the control group received sham acupuncture (CG). Both groups received antidepressants with adjunctive medication (i.e., benzodiazepines, tricyclics, or antipsychotics) as the standard treatment. All participants were assessed for executive functions and memory using specific cognitive tests, including the Trail Making Test B (TMT-B), Stroop Color and Word Test (SCWT), category delayed recall in the Alzheimer’s Disease Assessment Scale–Cognitive Subscale (ADAS-Cog), ^19^^,^^20^ and subjective reports of concern regarding concentration, memory, problem-solving, learning, communication, and quality of life (QoL) concerns using the WHO Disability Assessment Schedule (WHODAS 2.0; sections D1.1-1.6 and H1-3). ^21^ Additionally, depressive symptoms were assessed using the Thai-PHQ-9. All administrative tasks and data entry were carried out by the research assistant, who was also not involved in the interventions and outcome assessment ( Figure 1A,B).

This study was double-blinded RCT. All participants and assessors were unaware of group assignments. Before treatment began, participants would not recognize if they were in the real acupuncture or sham group. They were placed in separate, quiet units on different days.

The elimination or termination criteria were: (i) participants whose depressive symptoms worsened and required different treatment during the trial, making them unfit to continue. (ii) Participants facing severe adverse events or complications during treatment. (iii) Participants choosing not to continue with the treatment.

### Interventions

This present study utilized applied electroacupuncture from traditional Chinese medicine. In the electroacupuncture group (EG), participants received acupuncture at 10 scalp points. These points included: 1) Baihui [GV20] and Ex-hn 1, with 5 needles inserted to a depth of 0.5 millimeters, 2) the 3 points of intelligence composed of Shenting [GV24] and Benshen [GB13] on both sides, with one needle in each point inserted to a depth of 2 centimeters, and 3) Touwei [ST8] on both sides, with one needle in each point inserted to a depth of 2 centimeters. Additionally, participants in the EG received acupuncture at Tai Chong [LV3], Tai Yuan [LU9], and Tai Xi [KI3] on both sides, totaling 6 needles, inserted to a depth of 2 centimeters, as shown in Figure 1. All acupuncture was administered without regard to De Qi and retained for 20 minutes.

The electroacupuncture in the EG was applied for 20 minutes at Benshen [GB13] and Touwei [ST8] on both sides. The needle stimulation was DC 9 volts with 50 Hertz continuous dense waves (less than 1 milliampere). The electroacupuncture sessions were weekly for 10 weeks. The needles were made from sterilized stainless steel with a size of 0.20×40 millimeters.

In the sham acupuncture control group (CG), participants received acupress or a brief needle insertion at He Gu with 2 needles inserted to a depth of 1.3 millimeters on both hands for 20 minutes. The needles were made from sterilized stainless steel with a size of 0.20×1.3 millimeters. The participants in CG received this sham acupuncture only twice, at the first and 10th weeks, marking the endpoint of the study.

Both participants in EG and CG received the interventions from the certified acupuncturist (Boontra Y.) from the Department of Thai Traditional and Alternative Medicine, and Hunan University of Chinese Medicine in 2016, with 5 years of experience.

### Outcome

The primary outcome measured specific cognitive functions including the TMT-B, SCWT, ADAS-Cog; category delayed recall; and subjective cognitive, and quality of life complaints from the WHODAS 2.0; sections D1.1-1.6 and H1-3, which is a questionnaire asking about the number of days in the past 30 days when participants experienced difficulties with attention, understanding, and initiating conversation. All of these outcomes were assessed by trained blinded psychologists. The scores from the test at week 10 (T10), which serves as the primary endpoint, were compared to the scores from the test at week 1 (T1) for each participant. Then, the median value of the within-group change scores was calculated. Secondary outcome measured depressive symptoms using the Thai-PHQ-9, which were self-rated by the participants. All treatment outcomes were assessed at baseline (T1) and after the 10-week intervention (T10).

### Statistic analysis

The data was presented as mean ± standard deviation and median, and analyzed using SPSS 22.0 software. ^22^ A comparison between the electroacupuncture and control groups was conducted using the Mann-Whitney U test, which compared the median differences of the outcomes. Differences between groups were calculated by comparing baseline and endpoint values. A significance level of p < 0.05 was used to determine statistical significance. To compare all outcomes for all participants who were randomized to electroacupuncture and control groups, an intention-to-treat (ITT) analysis was performed with the last observation carried forward technique (LOCF). A per-protocol (PP) analysis was also performed for comparison among participants who completed the treatment.

## Results

### Demographic characteristics

**Figure 2.**
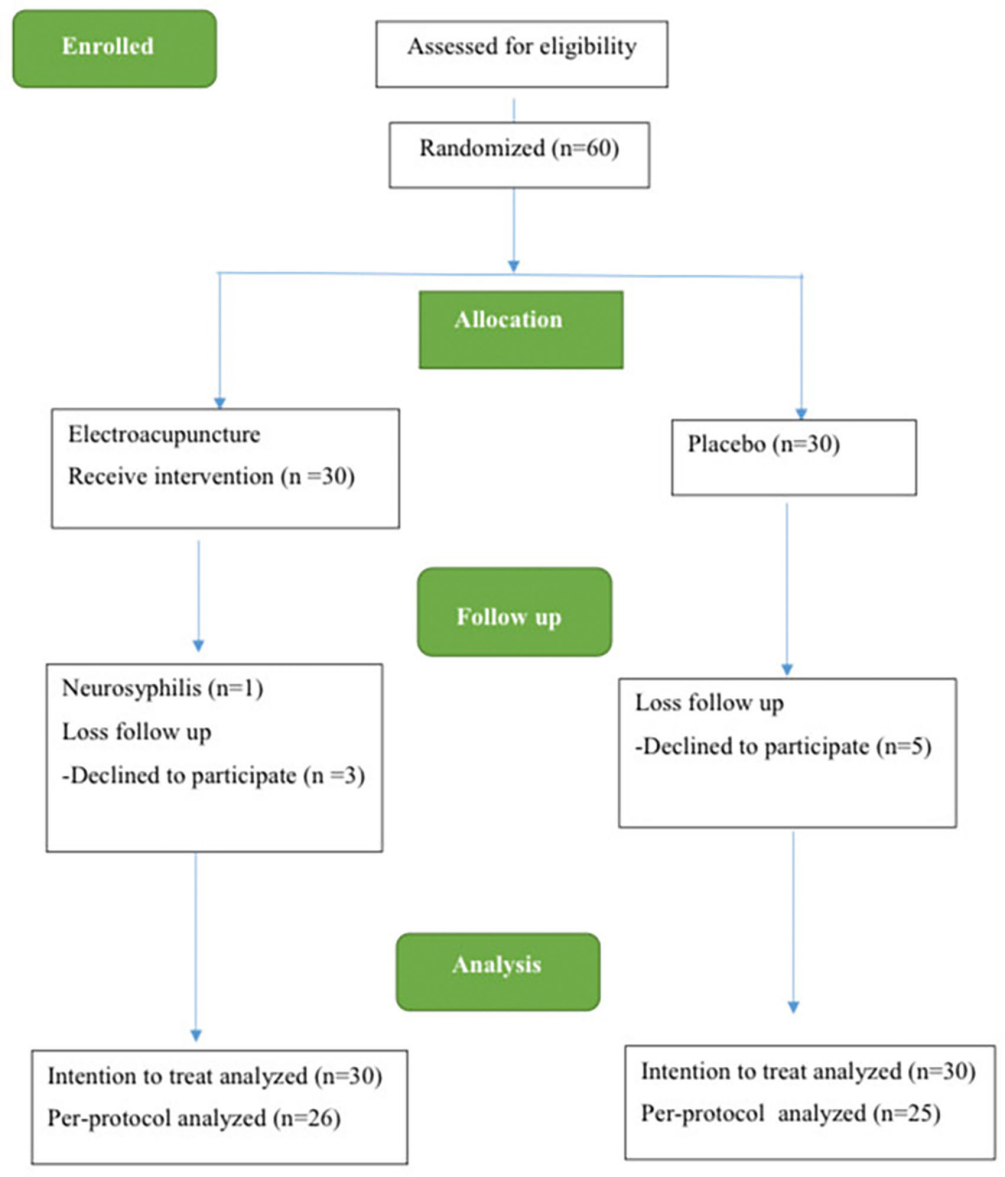
The CONSORT flow diagram.

The study spanned from December 2021 to December 2022. Initially, 60 individuals with MDD were enlisted for the trial. However, 8 participants, including 3 participants from the acupuncture group and 5 participants from the control group, discontinued their involvement during this period due to loss of follow-up. Additionally, 1 participant in the electroacupuncture group withdrew due to a neurosyphilis diagnosis. Consequently, 60 participants were eligible to receive ITT analysis, while 51 patients formed the complete analysis group using PP analysis ( Figure 2).

In terms of general characteristics (such as gender, age, education, underlying medical conditions, occupation, and severity of illness) of EG and CG were similar, the outcomes were analyzed with unadjusted estimates. Both sample groups are predominantly female, with an average education duration of approximately 15 years. The average age in the experimental group is 28.77 ± 9.16 years, while the average age of the control group is 27.57 ± 8.08 years. Most participants in both the experimental and control groups are employed and do not have pre-existing illnesses. In terms of clinical data, it was found that participants in both the experimental and control groups had similar durations of experiencing depressive symptoms (experimental group 3.3 ± 1.87 years, control group 3.7 ± 2.67 years). The Thai-PHQ-9 scores indicated a similar severity of depressive symptoms in both groups (experimental group 14.53 ± 4.96, control group 15 ± 4.15). Additionally, the average scores of WHODAS 2.0 D1.1-1.6, representing the level of severity in attention and memory-related life impairments, were similar between the two groups ( Table 1).

**Table 1.** Demographic data.

Variables	EG (n = 30)		CG (n = 30)	
*Gender*				
Female, n (%)	23	(76.3)	22	(73.3)
Male, n (%)	7	(27.6)	8	(26.7)
*Age* (year), mean (SD)	28.77	(9.16)	27.57	(8.08)
*Age* (year), min. - max.	20-49		18-50	
*Education* (year), mean (SD)	15.87	(1.83)	14.50	(2.47)
Education (year), min. - max.	9-18		8-16	
*Underlying*, n (%)	20	(66.7)	25	(83.3)
- DM	2	(6.7)	0	(0)
- HT	2	(6.7)	0	(0)
- Dyslipidemia	1	(3.3)	0	(0)
- Cardiovascular disease	0	(0)	0	(0)
- Other*	8	(26.7)	5	(16.7)
*Occupation,* n (%)				
- Employed	15	(50.0)	21	(70.0)
- Student	11	(36.7)	8	(26.7)
- unemployed	4	(13.3)	1	(3.3)
*Depression* (Thai PHQ-9), mean (SD)	14.53	(4.96)	15	(4.15)
*Depression* (Thai PHQ-9), min.-max.	3-19		4-19	
*Duration of illness* (year), mean (SD)	3.3	(1.87)	3.7	(2.67)
*Duration of illness (year), * min.-max.	0.6-8.2		0.5-14.0	
*Subjective cognitive complaints, * mean (SD)				
- WHODAS 2.0 (D1.1-1.6)	17.73	(4.66)	16.93	(3.78)
min. - max.	8-27		9-24	
- WHODAS 2.0 H1	19.47	(8.49)	17.33	(7.91)
min. - max.	5-30		3-30	
- WHODAS 2.0 H2	7.80	(8.13)	8.20	(8.89)
min. - max.	0-30		0-30	
- WHODAS 2.0H3	11.27	(10.05)	9.23	(9.04)
min. - max.	0-30		0-30	
*Average number of treatments*, mean (SD)	8.53	(2.70)	1.83	(0.379)
*Average number of treatments*, min. - max.	1-10		1-2	

Moreover, most participants used medication, primarily SSRIs, and sometimes benzodiazepines in a similar manner (see Table 2).

**Table 2.** Pharmacotherapy for major depressive disorder.

Medication	EG (n=30) n (%)	CG (n=30) n (%)
Selective Serotonin Reuptake inhibitor (SSRI)	23 (76.7)	27 (90.0)
- Sertraline	14	18
- Fluoxetine	8	7
- Escitalopram	1	3
Serotonin and Norepinephrine Reuptake Inhibitor (SNRI)	6 (20.0)	3 (10.0)
- Venlafaxine	6	3
Other antidepressants		
- Trazodone	4	3
- Bupropion	2	0
- Mirtazapine	1	0
- Vortioxetine	1	1
- Agomelatine	0	1
Tricyclic antidepressant (TCA)		
- Amitriptyline	4	1
- Nortriptyline	6	3
Antipsychotic		
- Quetiapine	3	2
- Risperidone	3	2
Benzodiazepines	28 (93.33)	24 (80)
- Lorazepam	17	14
- Clonazepam	9	7
- Clorazepate	2	3

**Note:** Some patients received multiple types of antidepressants concurrently or in combination with other drug classes such as benzodiazepines or antipsychotics. Some patients did not receive SSRIs as the primary medication but were prescribed other types of antidepressants. The medication administration followed the guidelines set by the American Psychological Association (2019) in the Clinical Practice Guideline for the Treatment of Depression Across Three Age Cohorts.

There is no use of medication such as citalopram, fluvoxamine, paroxetine, desvenlafaxine, duloxetine, levomilnacipran, imipramine, doxepin, clomipramine, aripiprazole, lithium, methylphenidate, thyroid hormone, diazepam, or alprazolam.

The assessment of executive function, memory, and subjective cognitive complaints at the baseline (T1) and endpoint (T10) of the study ( Table 3), which was conducted at the 10th week for both the electroacupuncture treatment group and the sham acupuncture control group, yielded the following results:

**Table 3.** Scores on assessment of executive function, subjective cognitive and quality of life.

Outcomes	EG (n=30)		CG (n=30)	
	mean (SD)	median	mean (SD)	median
Main outcome				
*Executive function: Trail making test B*				
- Trail making test B T1	131.67 (57.79)	112.50	146.60 (59.80)	132.50
- Trail making test B T10	119.87 (92.12)	94	120.20 (58.98)	119.50
*Executive function (inhibition capacity): Stroop test*				
- Stroop word test T1	61.40 (9.60)	63.00	63.14 (14.68)	65.50
- Stroop word test T10	68.63 (9.44)	68.50	68.50 (12.73)	64.50
- Stroop color test T1	78.73 (15.52)	80.50	80.13 (16.62)	82.00
- Stroop color test T10	89.43 (10.65)	93.00	87.43 (14.50)	92.00
- Stroop word and color test T1	33.7 (8.64)	34.50	34.90 (9.27)	34.50
- Stroop word and color test T10	39.53 (8.02)	40	36.73 (10.08)	35.5
*Memory*				
ADAS-cog delayed recall memory T1	7.43 (1.96)	8.00	7.53 (1.75)	7.50
ADAS-cog delayed recall memory T10	8.80 (1.12)	9.00	8.67 (1.56)	9.00
*Subjective cognitive complain*				
WHODAS 2.0 D1.1-1.6 T1	17.73 (4.66)	18.50	16.93 (3.78)	17.00
WHODAS 2.0 D1.1-1.6 T10	13.43 (3.87)	13.00	15.47 (3.37)	15.50
WHODAS 2.0 H1 T1	19.47 (8.49)	20.00	17.33 (7.91)	20.00
WHODAS 2.0 H1 T10	11.97 (10.09)	8.50	13.87 (9.19)	10.00
WHODAS 2.0 H2	7.80 (8.13)	5	8.20 (8.89)	4.50
WHODAS 2.0 H2 T10	4.47 (7.08)	2.00	4.77 (7.29)	2.00
WHODAS 2.0 H3 T1	11.27 (10.05)	8.50	9.23 (9.06)	6.00
WHODAS 2.0 H3 T10	7.97 (9.14)	3.00	8.50 (8.89)	5.00
*Secondary outcome depression*				
PHQ-9 T1	14.53 (4.96)	16.50	15.00 (4.15)	16.00
PHQ-9 T10	9.90 (5.81)	9.50	13.10 (5.65)	11.50

Note: T1 = baseline at week 1, T10 = week 10.

Scoring for the Trail Making B Test, WHODAS 2.0 (D1.1-D1.6 and H1-H3), and PHQ-9 uses reversal scoring, where lower scores indicate higher capability.

WHODAS 2.0 Thai version (D1.1-D1.6): In the past 30 days, difficulties in concentration, problem-solving, understanding, and initiating conversations.

H1: In the past 30 days, how many days did you experience problems?

H2: In the past 30 days, how many days were you unable to perform some activities due to health problems?

H3: In the past 30 days, how many days did you have to cut down or reduce activities due to health problems?

PHQ-9: The Nine-Item Patient Health Questionnaire for depression, Thai version.

Since the data distribution is not normal, the Independent Samples Mann-Whitney U Test was used to compare differences, with a statistical significance level of 0.05.

In the electroacupuncture treatment group ( Table 4), EG, executive function was primarily evaluated using the TMT- B. The average score of the TMT-B at T1 was 131.67 and at T10 was 119.87, indicating a trend of decreasing scores after receiving electroacupuncture. Subcomponents of executive function, such as inhibition capacity, were assessed using neuropsychological battery scores from the Stroop Word Test, Stroop Color Test, Stroop Word-Color Test, and memory scores from the ADAS-Cog Delayed Recall Memory test. The average scores for these subcomponents showed a tendency to improve after receiving electroacupuncture. Additionally, subjective cognitive complaints were evaluated using the Thai version of the WHODAS 2.0: D1.1-1.6. It was observed that there was a reduction in scores, with the average WHODAS 2.0: D1.1-1.6 score at T1 being 17.73 and at T10 being 13.43, suggesting a decrease in cognitive complaints. Furthermore, the secondary outcome, which measured the severity of depressive symptoms using the Thai-PHQ-9, also showed a similar trend of reduction.

**Table 4.** Treatment effects of electroacupuncture (analyses for intention-to-treat using the last observational carried forward method).

Outcomes	∆ median		Mann-Whitney U test	P-value
	EG (n=30)	CG (n=30)		
Main outcome				
*Executive function:*				
∆Trail making test B T10-T1	-15.00	-21.50	410.00	0.553
∆Stroop word test T10-T1	8.00	3.00	366.50	0.216
∆Stroop color test T10-T1	8.50	0.50	826.50	0.184
∆Stroop word and color test T10-T1	4.00	1.50	326.00	0.064
*Memory*				
∆ADAS-cog delay recall memory T10-T1	1.50	1.00	403.50	0.476
*Subjective cognitive complaint*				
∆WHODAS 2.0 D1.1-1.6 T10-T1	-5.50	0.00	318.00	0.049
∆WHODAS 2.0 H1 T10-T1	-5.50	-2	354.50	0.154
∆WHODAS 2.0 H2 T10-T1	0.00	-2	435.50	0.828
∆WHODAS 2.0 H3 T10-T1	-1.00	0.00	390.00	0.370
*Secondary outcome: depression*				
∆PHQ-9 T10-T1	-1	-1	331.00	0.077

Notes: T1 = baseline at week 1, T10 = week 10.

Scoring for the Trail Making B Test, WHODAS 2.0 (D1.1-D1.6 and H1-H3), and PHQ-9 uses reversal scoring, where lower scores indicate higher capability.

WHODAS 2.0 Thai version (D1.1-D1.6): In the past 30 days, difficulties in concentration, problem-solving, understanding, and initiating conversations.

H1: In the past 30 days, how many days did you experience problems?

H2: In the past 30 days, how many days were you unable to perform some activities due to health problems?

H3: In the past 30 days, how many days did you have to cut down or reduce activities due to health problems?

PHQ-9: The Nine-Item Patient Health Questionnaire for depression, Thai version.

Since the data distribution is not normal, the Independent Samples Mann-Whitney U Test was used to compare differences, with a statistical significance level of 0.05.

In the sham acupuncture control group, CG, the assessment of executive function using the TMT-B resulted in an average score of 146.60 at T1 and 120.20 at T10, indicating a trend of decreasing scores after receiving sham acupuncture. Similar to the electroacupuncture group, subcomponent assessments of executive function and memory showed a tendency to increase after receiving sham acupuncture. Subjective cognitive complaints, evaluated using the WHODAS 2.0: D1.1-1.6, also showed a decrease in scores, with the average WHODAS 2.0: D1.1-1.6 score at T1 being 16.93 and at T10 being 15.47, indicating a reduction in cognitive complaints. The secondary outcome, which measured the severity of depressive symptoms using the Thai-PHQ-9, showed a trend of reduction as well. The average Thai-PHQ-9 score at T1 was 15.00, and at T10 it was 13.10.

The difference in median scores between week 10 and week 1 in each group, measured using the TMT-B (which assesses flexibility and processing speed in terms of time in seconds), ADAS-cog delayed recall memory (used to evaluate cognitive function in memory), and Stroop Test (which has 3 subparts: Stroop Word Test, Stroop Color Test, and Stroop Word and Color; with Stroop Word and Color representing inhibition capacity), Thai-PHQ-9 (for measure depressive symptom) showed a trend of higher scores in the electroacupuncture group compared to the control group. However, when subjected to the Mann-Whitney U test, no statistically significant difference was found.

The analysis revealed that in the electroacupuncture group, the median within-group change score (∆WHODAS 2.0: D1.1-1.6 of T10-T1) was -5.50, indicating a significant improvement in difficulties with attention, understanding, and initiating conversation. On the other hand, in the control group, the median within-group change score (∆WHODAS 2.0: D1.1-1.6 of T10-T1) showed no change at 0 points. The Mann-Whitney U test was performed with a value of 318.00 and a corresponding p-value of 0.049, indicating a statistically significant difference between the experimental and control groups. This suggests that the experimental group experienced a statically significant decrease in difficulties with attention, understanding, and initiating conversation compared to the control group. No severe side effects were observed from the acupuncture treatment, such as nerve injury, internal organ injury, infection, allergies, or adverse reactions.

## Discussion

Findings from the study indicate that electroacupuncture can statically significantly reduce subjective cognitive complaints in individuals with mild to moderate depressive symptoms and cognitive issues, when compared to the control group. These complaints encompass difficulties in activities like concentration, thinking, analysis, learning, understanding, and initiating conversations over the past 30 days.

Moreover, positive trends were observed in the experimental group, showcasing enhancements in executive function, memory, and a reduction in the frequency and severity of cognitive issues and depressive symptoms. These outcomes might partly arise from the placebo response, where individuals in the control group experience health improvements despite receiving a simulated treatment. This phenomenon is influenced by participants’ perception of the experiment, including their emotions, expectations, and the therapeutic environment. Interestingly, placebo effects can also be seen in the active treatment group.

A meta-analysis by Hafliðadóttir, S.H., and colleagues spanning from 1966 to 2008 covering randomized controlled trials, highlighted that contextual effects were notably higher when the outcome assessor was blinded and the allocation was concealed. Such effects were more pronounced in younger patients and with a higher proportion of female participants. ^23^ In our study, the authors were mindful of the potential for placebo responses and took measures to minimize them, both for participants and assessors.

Despite the predominance of female participants in our study, both groups displayed improvements. Notably, the experimental group exhibited significantly greater enhancements in subjective cognitive complaints. This could be attributed to the experimental group receiving more frequent acupuncture interventions, resulting in a more pronounced contextual effect (around 8.53 times) compared to the control group (approximately 1.83 times). However, even though the researcher tried to design sham acupuncture using very small and short needles, believing they might not have any effect at the acupuncture points, the points used were He gu points, which could influence the placebo effect. Therefore, designing sham acupuncture should avoid using actual acupuncture points, as this may better the differences between the two groups.

Acupuncture, compared to non-invasive brain stimulation, stands out as a cost-effective, easily accessible treatment with minimal side effects. It brings about an enhanced quality of life for participants, evident from reduced subjective cognitive complaints in both experimental and control groups. This reduction underscores acupuncture’s positive influence.

As participants experience relief in subjective cognitive complaints, their apprehensions about resuming daily activities diminish. Worries regarding work capacity decrease, leading to enhanced work efficiency through reduced anxiety and an improved overall quality of life. Positive developments were also noted in other aspects, such as executive function, memory, motor speed, and inhibition. Moreover, acupuncture shows potential to lower the recurrence rate of depressive symptoms.

Previous research has shown that acupuncture improves cognitive function. For example, Jinyu Du, et al. studied the clinical effect of scalp acupuncture combined with cognitive training on cognitive impairment after cerebral injury, comparing it to receiving routine treatment and cognitive rehabilitation training for 12 weeks. They found that the scores of cognitive function assessment in both groups were significantly higher than before treatment. Additionally, in the three cognitive sub-domains—orientation, visual motor organization, and thinking operation—the scores of the treatment group were significantly higher than those of the control group. ^8^

In another study by Zuo-Li Sun, et al., focusing on the efficacy of electroacupuncture (EA) in patients with schizophrenia, it was found that EA significantly improved memory and moderately improved executive functions and problem-solving. However, it did not demonstrate significant improvement in the severity of psychiatric symptoms or BDNF levels between the control and experimental groups. ^9^

Yujie Jia, et al. conducted a study on the efficacy of acupuncture with manual needle stimulation in patients with mild to moderate Alzheimer’s disease. They found that the group receiving acupuncture with manual needle stimulation showed significant benefits in cognitive functions measured by the ADAS-cog score compared to the group receiving only Donepezil. However, there was no significant difference in activities of daily living between the two patient groups. ^24^

Notably, successful treatments in those studies involved regular acupuncture on alternate days. Literature review by the researchers spotlighted the dose-dependent effect of acupuncture treatment, categorized as low and high dose. High dose acupuncture exhibits the following attributes: 1) using over 9 needles, 2) administering treatment more than twice weekly, 3) generating a “De qi” sensation with at least 8 needle insertions, and 4) displaying strong positive correlation with treatment outcomes.

Prior high dose acupuncture studies involved daily treatment for at least 2 weeks, more than 10 needle insertions, and over 9 needles. In contrast, this study employed once-weekly treatment, over 9 needle insertions (at least 6 lacking the “De qi” sensation at each insertion), not qualifying as high dose acupuncture. Limited participant numbers (n=30) due to time and resource constraints might have contributed to statistically insignificant findings.

Cognitive impairment in patients with depressive disorder is linked to inflammation. When inflammation occurs in the central nervous system, known as neuroinflammation, it triggers the production of pro-inflammatory cytokines, tumor necrosis factor-a (TNF-a), chemokines, small-molecule messengers such as prostaglandins and nitric oxide (NO), as well as reactive oxygen species from innate immune cells in the CNS. This oxidative stress is associated with damage to DNA, RNA, lipids, and proteins, with pyramidal neurons in the hippocampus being particularly sensitive to energy deficits. This vulnerability contributes to cognitive impairment. Moreover, oxidative stress leads to a decrease in the expression and function of tropomyosin receptor kinase B (Trk B), a receptor that is crucial for brain-derived neurotrophic factor (BDNF), which plays an essential role in promoting brain plasticity. ^25^^,^^26^

Due to its ability to stimulate the expression of GSK-3β/PP2A, acupuncture can enhance neurogenesis in the hippocampus. This results in improved cerebral blood flow in the ischemic cortex, hippocampus, and striatum. Additionally, acupuncture increases the levels of neurotrophic factors such as BDNF and VEGF (brain-derived neurotrophic factor and vascular endothelial growth factor). BDNF and VEGF play crucial roles in stimulating neurogenesis, promoting the formation of new neurons, and facilitating neuronal migration in neurogenic areas. Moreover, VEGF itself is a significant contributor to the angiogenic process. ^12^

During ischemic conditions in the brain, the body naturally responds by promoting the formation of new blood vessels, serving as an essential neuroprotective factor. ^27^ Electroacupuncture has been shown to stimulate the release of VEGF, which can be detected in peripheral blood, and to increase cerebral blood flow and cerebral blood volume. This also improves motor function in patients with ischemic stroke. ^13^ Additionally, experiments on rats have demonstrated that acupuncture can stimulate the release of nitric oxide (NO) and reduce the release of angiotensin II, leading to greater dilation of cerebral blood vessels. ^14^^,^^15^

When the release of these substances is stimulated, it is found that the half-life of BDNF in the brain is unknown. However, the half-life of nerve growth factor (NGF), a neurotrophin of similar size and charge, is reported to be less than one hour. ^28^ Since BDNF and NGF both activate the Trk family of receptors, it is likely that the half-life of BDNF is also on the order of hours. Therefore, electroacupuncture should be administered relatively frequently to maintain effective levels of BDNF or VEGF. It is recommended to apply the treatment at a high dose, which means more than twice a week. This may explain why the researcher's study did not yield significant results.

Furthermore, no previous research focused on acupuncture for depressive disorder patients with cognitive dysfunction, limiting direct comparison of acupuncture techniques.

Depression patients often grapple with cognitive problems, attention, and memory issues. These difficulties often persist even after depressive symptoms alleviate, increasing the risk of mild cognitive impairment (MCI) or Alzheimer’s disease. Acknowledging, diagnosing, and continually monitoring subjective cognitive decline in depressed patients are crucial for proper treatment and follow-up. In this study, interpreting the findings that electroacupuncture potentially reduces subjective cognitive complaints in MDD patients with mild to moderate depressive symptoms and cognitive issues, compared to the control group, should take into account three considerations: firstly, the baseline subjective cognitive assessment in the control group were less severe than those in the experimental group; and secondly, the ceiling effect, as the baseline scores of executive function, subjective cognitive complaints, and quality of life were relatively high in both groups; and thirdly, in the sham group, although the authors believe that the very short length of the needle without De Qi and the small dose of acupuncture may not be effective, future research should use non-acupuncture points to reduce confounding factors.

Addressing these concerns, the authors conducted a pilot study suggesting that electroacupuncture may enhance cognitive function more than the control group. These findings pave the way for further research, both clinically and potentially in clinics. Extended studies are proposed, involving non-invasive transcranial stimulation or electroacupuncture with higher doses, frequency, and duration. Additionally, specific acupuncture points correlating with brain function and a larger participant pool could yield clearer effect sizes and concrete evidence, fostering novel knowledge and pioneering treatments for improved mental health services in the future.

### Limitation

This present study was carried out within a specific timeframe, from December 24, 2021, to December 23, 2022, and focused solely on depression patients seeking treatment at Thammasat University Hospital. Consequently, participant numbers were limited, and the study faced constraints due to outpatient treatment frequency. Participants were unable to undergo acupuncture more than once weekly, categorizing the treatment dose as low. As a result, the study’s outcomes might not distinctly exhibit differences.

Furthermore, findings from the present study indicate that electroacupuncture might be able to alleviate subjective cognitive complaints in individuals with mild to moderate depressive symptoms and cognitive issues, compared to the control group. However, the clinical significance of the outcomes of electroacupuncture remains unclear.

The authors did not include a healthy control group, focusing instead on patients with MDD who had subjective complaints and were receiving standard pharmacological treatment. The present study aimed to see how these patients responded to adjunctive electroacupuncture. Patients with MDD often receive various psychotropic drugs, such as benzodiazepines and antipsychotics, which can affect cognitive function and serve as confounding factors. Previous research suggested that acupuncture can influence cognitive function, so the study focused on its use as an adjunctive treatment in real-world settings.

Future studies may include a much larger number of patients with these characteristics to detect conclusive differences between groups, or in a smaller study, limit acupuncture as an augmentation of one specific class of medications.

## Ethics and consent

The Human Research Ethics Committee of Thammasat University (Medicine) has granted approval for this medical research study (MTU-EC-PS-1-304/64) with an approval date from December 24, 2021 to December 23, 2022. It is in full compliance with international guidelines such as the Declaration of Helsinki. Each participant was provided with detailed information regarding the study and informed written consent was obtained for their participation in the research. Subsequently, all participants granted consent for publication.

## Data Availability

Zenodo: Effects of Electroacupuncture on Cognitive Symptoms in Major Depressive Disorder: A Pilot Study and Randomized Controlled Trial, https://doi.org/10.5281/zenodo.10488186. ^29^ Zenodo: Checklist for Effects of Electroacupuncture on Cognitive Symptoms in Major Depressive Disorder: A Pilot Study and Randomized Controlled Trial, https://doi.org/10.5281/zenodo.10816401. ^30^ Data are available under the terms of the Creative Commons Attribution 4.0 International license (CC-BY 4.0). Zenodo: STRICTA 2010 checklist for Effects of Electroacupuncture on Cognitive Symptoms in Major Depressive Disorder: A Pilot Study and Randomized Controlled Trial, https://doi.org/10.5281/zenodo.12515179. Data are available under the terms of the Creative Commons Attribution 4.0 International license (CC-BY 4.0).
