# Supplementary material for: Effects of electroacupuncture on cognitive symptoms in major depressive disorder: a pilot study and randomized controlled trial
Source: F1000Res. 2024 Dec 23;13:479. Originally published 2024 May 17. [Version 4] doi: 10.12688/f1000research.146897.4 (PMC11605169; doi:10.12688/f1000research.146897.4)
Supplement: Peer review history

# Reviewer report: Reviewer response for version 3

**DOI:** 10.5256/f1000research.170190.r326928

**Published:** 2024-11-28

**Authors:** Min Cai

**Affiliations:** 1 The Fourth Military Medical University, Shaanxi, China

In the manuscript titled “Effects of electroacupuncture on cognitive symptoms in major depressive disorder: a pilot study and randomized controlled trial”, Yindee Boontra et al. reported a randomized controlled trial to investigate the role of EA on cognitive symptoms and quality of life in patients suffered from MDD. Some concerns still need to be issued before the conclusion can be got.

 1.    About the trial registration of this study, I noted that the authors registered this trial at February 2, 2024, however, they started to enrolled participants from December 24, 2021. This is totally not be approved in current ethic protocol. 

 2.    About the sample size, how the authors calculated the sample size? In according the previous study, or just set as a pilot study? I think a scientific hypothesis should be added to identify the current sample size is enough to get the conclusion. 

 3.    How to identify the cognitive dysfunction in MDD patients? The criteria should be issued in the revised manuscript. 

 4.    How the double blinded protocol work? I noticed that the real EA treatment protocol and the sham one are totally different, so how the authors blind the operation during the experiment should be calefied

 5.    About the discussion section, some citations are focused on cerebral ischemia, I think more citations in relevance of cognitive dysfunction should be citated.

Is the work clearly and accurately presented and does it cite the current literature?

No

If applicable, is the statistical analysis and its interpretation appropriate?

I cannot comment. A qualified statistician is required.

Are all the source data underlying the results available to ensure full reproducibility?

Yes

Is the study design appropriate and is the work technically sound?

Partly

Are the conclusions drawn adequately supported by the results?

No

Are sufficient details of methods and analysis provided to allow replication by others?

Partly

Reviewer Expertise:

NA

I confirm that I have read this submission and believe that I have an appropriate level of expertise to confirm that it is of an acceptable scientific standard, however I have significant reservations, as outlined above.

# Response

**Published:** 2024-12-16

**Authors:** Muthita Phanasathit

**Affiliations:** Department of Psychiatry, Thammasat University, Pathum Thani, Thailand

**About the trial registration of this study, I noted that the authors registered this trial at February 2, 2024, however, they started to enrolled participants from December 24, 2021. This is totally not be approved in current ethic protocol.**

           Thank you for your valuable feedback regarding the ethics committee approval for our study. We would like to clarify that our research was approved by the Human Research Ethics Committee of Thammasat University (Medicine) on December 24, 2021 (MTU-EC-PS-1-304/64), prior to the data collection phase. This approval adhered to international ethical standards.

 Regarding the registration of our clinical trial to enhance research transparency and public accessibility, the authors also registered the study in the Thai Clinical Trials Registry (www.clinicaltrials.in.th) with the identifier NCT06239740, which was certified on February 2, 2024.

**The Manuscript was change from** : (First paragraph of Methods)  This trial was registered to the ClinicalTrials.gov (NCT06239740), with a registration date of February 2, 2024 and the Human Research Ethics Committee of Thammasat University (Medicine) has granted approval for this medical research study (MTU-EC-PS-1-304/64) with an approval date from December 24, 2021 to December 23, 2022. It is in full compliance with international guidelines such as the Declaration of Helsinki, The Belmont Report, CIOMS Guidelines, and the International Conference on Harmonisation-Good Clinical Practice (ICH-GCP). This study protocol outlined a double-blinded randomized controlled clinical trial (RCT) and pilot study design conducted at a single center, focusing on evaluating the impact of electroacupuncture on specific cognitive functions in individuals with major depressive disorder (MDD) who experience cognitive dysfunction when compared to the control group, while both experimental and control groups were under the standard pharmacological treatment for depressive disorder. The present study was conducted during the Coronavirus Disease (COVID-19) pandemic. The study was adapted to a pilot study with 30 samples per group due to safety concerns and social distancing measures. The preliminary outcomes aim to inform and guide future main research on the effects of electroacupuncture on cognitive dysfunction in MDD patients 

**To this**

         This trial was registered with the Human Research Ethics Committee of Thammasat University (Medicine), which approved the study (approval code: MTU-EC-PS-1-304/64) for the period from December 24, 2021, to December 23, 2022. The study adhered fully to international ethical guidelines, including the Declaration of Helsinki, The Belmont Report, CIOMS Guidelines, and the International Conference on Harmonisation-Good Clinical Practice (ICH-GCP). The research protocol detailed a double-blinded, randomized controlled trial (RCT) and pilot study conducted at a single center. The study aimed to evaluate the impact of electroacupuncture on specific cognitive functions in individuals with major depressive disorder (MDD) and cognitive dysfunction, compared to a control group. Both the experimental and control groups received standard pharmacological treatment for depressive disorders. This study was conducted during the Coronavirus Disease (COVID-19) pandemic, necessitating modifications due to safety concerns and social distancing measures. As a result, the study was adapted to a pilot design with 30 participants in each group. The preliminary findings are intended to guide and inform future large-scale research on the effects of electroacupuncture on cognitive dysfunction in patients with MDD. To enhance research transparency and public accessibility, the authors registered the study with the Thai Clinical Trials Registry (www.clinicaltrials.in.th), under the identifier NCT06239740, certified on February 2, 2024.

**2.    About the sample size, how the authors calculated the sample size? In according the previous study, or just set as a pilot study? I think a scientific hypothesis should be added to identify the current sample size is enough to get the conclusion.**

**Answer:** The authors have titled their research *"Effects of Electroacupuncture on Cognitive Symptoms in Major Depressive Disorder: A Pilot Study and Randomized Controlled Trial" *and have provided details regarding the sample size in the Methods section as follows: *"The study was adapted to a pilot study with 30 samples per group due to safety concerns and social distancing measures. The preliminary outcomes aim to inform and guide future main research on the effects of electroacupuncture on cognitive dysfunction in MDD patients."*

              Initially, the study was designed as a randomized controlled trial (RCT) with a larger sample size, calculated based on statistical power analyses derived from previous studies on similar interventions for cognitive dysfunction in major depressive disorder (MDD). However, due to the unforeseen challenges posed by the COVID-19 pandemic, including safety concerns and the necessity for social distancing, the study was adapted to a pilot study with 30 participants per group. This adjusted sample size is intended to provide preliminary estimates of effect size and variability, which are crucial for designing adequately powered studies in the future. As stated by Totton, N., Lin, J., Julious, S. et al. in their review "A Review of Sample Sizes for UK Pilot and Feasibility Studies on the ISRCTN Registry from 2013 to 2020" (Pilot Feasibility Stud 9, 188, 2023; https://doi.org/10.1186/s40814-023-01416-w), the median sample size per arm in pilot and feasibility studies from 2013 to 2020 was found to be 30 participants.

**3.    How to identify the cognitive dysfunction in MDD patients? The criteria should be issued in the revised manuscript.**

 Answer: In our study, we used the PHQ-9 to assess subjective cognitive complaints, specifically focusing on item 7, which inquires about difficulties related to concentration and memory. Item 7 asks, "Trouble concentrating on things, such as reading the newspaper or watching television." The authors have revised and added these details in the manuscript.

**The manuscript was changed from** A total of 60 eligible participants, aged between 18 and 55 years, with cognitive symptoms resulting from MDD, were recruited from the psychiatry outpatient clinic at Thammasat University Hospital. All participants with MDD and low suicidal risk, diagnosed according to the DSM-516 or DSM-IV-TR criteria,17 and currently receiving standard treatment for at least 3 months from psychiatrists and psychiatric residents, were included. The subjective cognitive symptom was screened using the 7th item of the Thai version of the Patient Health Questionnaire (Thai-PHQ-9),18 which assesses experiencing trouble concentrating or cognitive problems (e.g., reading or watching TV).

**To this**: A total of 60 eligible participants, aged between 18 and 55 years, with cognitive symptoms resulting from MDD, were recruited from the psychiatry outpatient clinic at Thammasat University Hospital. All participants with MDD and low suicidal risk, diagnosed according to the DSM-5 ^16 ^or DSM-IV-TR criteria ^17^, and currently receiving standard treatment for at least 3 months from psychiatrists and psychiatric residents, were included. The subjective cognitive symptoms were screened using item 7 of the Thai version of the Patient Health Questionnaire (Thai-PHQ-9) ^18^, which assesses difficulties with concentration or cognitive problems. The question in item 7 reads, "Trouble concentrating on things, such as reading the newspaper or watching television."

**4.    How the double blinded protocol work? I noticed that the real EA treatment protocol and the sham one are totally different, so how the authors blind the operation during the experiment should be calefied**

         The details of the double-blinded protocol are provided in the Methods section as follows: "This study was a double-blinded RCT. All participants and assessors were unaware of group assignments. Before treatment began, participants were unable to distinguish whether they were in the real acupuncture or sham group. They were placed in separate, quiet units on different days."

 In detail, to prevent any interaction between the experimental and control groups, their appointments were scheduled on different days. The control group was only required to attend the initial session and the follow-up at week 10, during which they were kept in a separate room from the experimental group. This scheduling strategy effectively minimized the possibility of participants from both groups meeting. In summary, the researchers intentionally designed the study to ensure that participants, assessors (who are psychologists), and researchers responsible for statistical data analysis were blinded.

**5.    About the discussion section, some citations are focused on cerebral ischemia, I think more citations in relevance of cognitive dysfunction should be citated.**

       The discussion  about cognitive dysfunction was added in the manuscript

  Cognitive impairment in patients with depressive disorder is linked to inflammation. When inflammation occurs in the central nervous system, known as neuroinflammation, it triggers the production of pro-inflammatory cytokines, tumor necrosis factor-a (TNF-a), chemokines, small-molecule messengers such as prostaglandins and nitric oxide (NO), as well as reactive oxygen species from innate immune cells in the CNS. This oxidative stress is associated with damage to DNA, RNA, lipids, and proteins, with pyramidal neurons in the hippocampus being particularly sensitive to energy deficits. This vulnerability contributes to cognitive impairment. Moreover, oxidative stress leads to a decrease in the expression and function of tropomyosin receptor kinase B (Trk B), a receptor that is crucial for brain-derived neurotrophic factor (BDNF), which plays an essential role in promoting brain plasticity.

# Reviewer report: Reviewer response for version 2

**DOI:** 10.5256/f1000research.168555.r297844

**Published:** 2024-07-18

**Authors:** Su-Xia Li

**Affiliations:** 1 National Institute on Drug Dependence and Beijing Key laboratory of Drug Dependence Research, Peking University, Beijing, China

I think that most of the reviewer's concerns have been addressed.

 As for the explanation for “Many measures of cognitive function did not differ between the two groups after 10 weeks of treatment. Is it because the settings of the sham group are unreasonable?”. Authors explanation is not reasonable. Firstly, the baseline subjective cognitive function in the control group were not less severe than those in the experimental group, it is comparable. Secondly, the idea of ceiling effect is also unreasonable and inappropriate. In fact, authors have not explained anything for this problem.

 Additionally, in the Discussion section, the author should also discuss in depth the reasons for the lack of improvement in all objective indicators.

 In the abstract, the author used different words to describe the drug treatment taken by the two groups of subjects. In the main text, the two groups were actually the same, and it was suggested to use the same words to describe them.

 In the Table 2, the number of patients in CG totally 27 who took SSRIs, Instead, the number of patients who took Sertraline, Fluoxetine and Escitalopram combined was 28

 In the Table 3, both EG (n=30) and CG (n=30) should be placed in the correct position, corresponding to their respective data.

Is the work clearly and accurately presented and does it cite the current literature?

Partly

If applicable, is the statistical analysis and its interpretation appropriate?

Partly

Are all the source data underlying the results available to ensure full reproducibility?

Partly

Is the study design appropriate and is the work technically sound?

Partly

Are the conclusions drawn adequately supported by the results?

No

Are sufficient details of methods and analysis provided to allow replication by others?

Partly

Reviewer Expertise:

Psychiatry，Depression

I confirm that I have read this submission and believe that I have an appropriate level of expertise to confirm that it is of an acceptable scientific standard, however I have significant reservations, as outlined above.

# Response

**Published:** 2024-08-08

**Authors:** Muthita Phanasathit

**Affiliations:** Department of Psychiatry, Thammasat University, Pathum Thani, Thailand

**Response to Reviewers**

**1.    Reviewer Comment**: "I think that most of the reviewer's concerns have been addressed. As for the explanation for ‘Many measures of cognitive function did not differ between the two groups after 10 weeks of treatment. Is it because the settings of the sham group are unreasonable?’"

**Response**: The authors believe that sham acupuncture lacks efficacy and, due to safety concerns, aim to minimize injury and unnecessary infection risks from acupuncture needle insertion at non-acupuncture points connected to EA through wires. Therefore, we have added the following explanation to the Discussion section:

*“However, even though the researcher tried to design sham acupuncture using very small and short needles, believing they might not have any effect at the acupuncture points, the points used were He gu points, which could influence the placebo effect. Therefore, designing sham acupuncture should avoid using actual acupuncture points, as this may better differentiate between the two groups.”*

**2.    Reviewer Comment**: "Additionally, in the Discussion section, the author should also discuss in depth the reasons for the lack of improvement in all objective indicators."

**Response**: The authors have expanded the Discussion section to provide a more in-depth explanation of the low-dose regimen and its effects on neurotropins, as follows:

 “ *Due to its ability to stimulate the expression of GSK-3β/PP2A, acupuncture can enhance neurogenesis in the hippocampus. This results in improved cerebral blood flow in the ischemic cortex, hippocampus, and striatum. Additionally, acupuncture increases the levels of neurotrophic factors such as BDNF and VEGF (brain-derived neurotrophic factor and vascular endothelial growth factor). BDNF and VEGF play crucial roles in stimulating neurogenesis, promoting the formation of new neurons, and facilitating neuronal migration in neurogenic areas. Moreover, VEGF itself is a significant contributor to the angiogenic process. [1]*

*During ischemic conditions in the brain, the body naturally responds by promoting the formation of new blood vessels, serving as an essential neuroprotective factor. [2] Electroacupuncture has been shown to stimulate the release of VEGF, which can be detected in peripheral blood, and to increase cerebral blood flow and cerebral blood volume. This also improves motor function in patients with ischemic stroke. [3] Additionally, experiments on rats have demonstrated that acupuncture can stimulate the release of nitric oxide (NO) and reduce the release of angiotensin II, leading to greater dilation of cerebral blood vessels. [4, 5]*

*When the release of these substances is stimulated, it is found that the half-life of BDNF in the brain is unknown. However, the half-life of nerve growth factor (NGF), a neurotrophin of similar size and charge, is reported to be less than one hour. [6] Since BDNF and NGF both activate the Trk family of receptors, it is likely that the half-life of BDNF is also on the order of hours. Therefore, electroacupuncture should be administered relatively frequently to maintain effective levels of BDNF or VEGF. It is recommended to apply the treatment at a high dose, which means more than twice a week. This may explain why the researcher's study did not yield significant results.*”

*References*:

 1.    Kim, Y.R., et al., Electroacupuncture promotes post-stroke functional recovery via enhancing endogenous neurogenesis in mouse focal cerebral ischemia. PLoS One, 2014. 9(2): p. e90000 DOI: 10.1371/journal.pone.0090000.

 2.    Vallon, M., et al., Developmental and pathological angiogenesis in the central nervous system. Cell Mol Life Sci, 2014. 71(18): p. 3489-506 DOI: 10.1007/s00018-014-1625-0.

 3.    Zheng, C., et al., A randomized controlled trial comparing electroacupuncture with manual acupuncture for motor function recovery after ischemic stroke. European Journal of Integrative Medicine, 2018. 22: p. 76-80.

 4.    Kim, J.H., et al., Electroacupuncture acutely improves cerebral blood flow and attenuates moderate ischemic injury via an endothelial mechanism in mice. PLoS One, 2013. 8(2): p. e56736 DOI: 10.1371/journal.pone.0056736.

 5.    Li, J., et al., Electroacupuncture improves cerebral blood flow and attenuates moderate ischemic injury via Angiotensin II its receptors-mediated mechanism in rats. BMC Complement Altern Med, 2014. 14: p. 441 DOI: 10.1186/1472-6882-14-441.

 6.    Krewson, C.E. and W.M. Saltzman, Transport and elimination of recombinant human NGF during long-term delivery to the brain. Brain Res, 1996. 727(1-2): p. 169-81 DOI: 10.1016/0006-8993(96)00378-2.

**3.    Reviewer Comment**: "In the abstract, the author used different words to describe the drug treatment taken by the two groups of subjects. In the main text, the two groups were actually the same, and it was suggested to use the same words to describe them."

**Response**: The authors have revised the abstract for consistency. It now reads:

 “Participants were divided into two groups: the electroacupuncture group combined with standard pharmacological treatment (EG; n=30) and the control group receiving standard pharmacological treatment with placebo acupuncture (CG; n=30).”

**4.    Reviewer Comment**: "In Table 2, the number of patients in CG totally 27 who took SSRIs. Instead, the number of patients who took Sertraline, Fluoxetine, and Escitalopram combined was 28."

**Response**: The authors have added the following note under Table 2 to clarify:

 “ *Note: Some patients received multiple types of antidepressants concurrently or in combination with other drug classes such as benzodiazepines or antipsychotics. Some patients did not receive SSRIs as the primary medication but were prescribed other types of antidepressants. The medication administration followed the guidelines set by the American Psychological Association (2019) in the Clinical Practice Guideline for the Treatment of Depression Across Three Age Cohorts.*”

**5.    Reviewer Comment**: "In Table 3, both EG (n=30) and CG (n=30) should be placed in the correct position, corresponding to their respective data."

**Response**: The authors have corrected the placement of EG (n=30) and CG (n=30) at the header of Table 3.

# Reviewer report: Reviewer response for version 2

**DOI:** 10.5256/f1000research.168555.r297843

**Published:** 2024-07-16

**Authors:** Rosa N Schnyer

**Affiliations:** 1 School of Nursing, The University of Texas, Austin, Texas, USA

Although most comments from the reviewers have been addressed, the authors still describe the study as a double-blind study. It is not, or rather, it is impossible to assess if it fact the double blind worked. Please add a clause to explain how blinding was neither assessed and may not have been necessarily accomplished.

 It is important not to claim that a study is double, unless this has been effectively established. The authors need to remove the double blind statement, and explain that they set to conduct a double-blind study but since blinding wasn't assessed they cannot be sure that acupuncturists were in fact blind. Blinding of the treatment providers is not essential to the conclusions of the study but a caveat needs to be included. Thank you

Is the work clearly and accurately presented and does it cite the current literature?

Partly

If applicable, is the statistical analysis and its interpretation appropriate?

I cannot comment. A qualified statistician is required.

Are all the source data underlying the results available to ensure full reproducibility?

No source data required

Is the study design appropriate and is the work technically sound?

Partly

Are the conclusions drawn adequately supported by the results?

No

Are sufficient details of methods and analysis provided to allow replication by others?

Partly

Reviewer Expertise:

Acupuncture, depression, pain, women's health

I confirm that I have read this submission and believe that I have an appropriate level of expertise to confirm that it is of an acceptable scientific standard.

# Reviewer report: Reviewer response for version 1

**DOI:** 10.5256/f1000research.161025.r282200

**Published:** 2024-06-11

**Authors:** Su-Xia Li

**Affiliations:** 1 National Institute on Drug Dependence and Beijing Key laboratory of Drug Dependence Research, Peking University, Beijing, China

1. The treatment plan for the two groups of patients in the Abstract is different from the description in the Methods section.

 2. A healthy control group should be included in the study protocol. On the one hand, does the cognitive function of patients with MDD differ from that of healthy controls at baseline. On the other hand, after treatment, whether the cognitive function of patients is restored, and how much differences between healthy controls and patients with depression. These can only be achieved in the presence of a healthy control group.

 3. For the electroacupuncture group, the authors needed to describe the entire treatment process in detail. Such as the duration of each treatment, how many days consecutive per week, the specific depth of the acupuncture therapy, and how strong the current intensity is?

 4. How does the Sham group work？The authors need to describe in detail the specific operation of the sham acupuncture control group, how long the acupuncture of He gu point lasted for each treatment, and how many consecutive days were in the first week and the 10th week. The specific depth of the acupuncture therapy, and how strong the current intensity is?

 5. For the comparison of the two groups of indicators at the end point, the existence of time effect should be considered. It is unreasonable and unscientific to use a simple *t*-test between two groups.

 6. Authors defined that at baseline is T0, at the ten-week intervention is T10. However, in Results section, baseline is described as T1. The author's description of the same event should be uniform.  

 7. Abbreviations in the table should be explained at the bottom of the table.

 8. Many measures of cognitive function did not differ between the two groups after 10 weeks of treatment. Is it because the settings of the sham group are unreasonable?

Is the work clearly and accurately presented and does it cite the current literature?

Partly

If applicable, is the statistical analysis and its interpretation appropriate?

Partly

Are all the source data underlying the results available to ensure full reproducibility?

Partly

Is the study design appropriate and is the work technically sound?

Partly

Are the conclusions drawn adequately supported by the results?

No

Are sufficient details of methods and analysis provided to allow replication by others?

Partly

Reviewer Expertise:

Psychiatry，Depression

I confirm that I have read this submission and believe that I have an appropriate level of expertise to state that I do not consider it to be of an acceptable scientific standard, for reasons outlined above.

# Response

**Published:** 2024-06-26

**Authors:** Muthita Phanasathit

**Affiliations:** Department of Psychiatry, Thammasat University, Pathum Thani, Thailand

- According to the review comment, the revision of abstract was made. Page 2 Line 4-8 in abstract

 -  Thank you for your advice. We agree that a healthy control should be included in experimental studies aiming to assess the safety and efficacy of new interventions and medications. However, a literature review on the use of electroacupuncture has provided evidence of its safety and efficacy in treating patients with schizophrenia, brain injury, and stroke who have cognitive impairment. The researchers aimed to study the effects of electroacupuncture on specific cognitive functions in subjects experiencing cognitive dysfunction associated with MDD. Including healthy controls without subjective cognitive complaints in this study might not address the research question adequately. Therefore, we recruited MDD patients with cognitive dysfunction receiving sham acupuncture and standard treatment as a comparator instead.

 - According to the review comment, the revision was made in the method / intervention session. (page 4, Line 12-31)

 - Due to the outcome from this pilot study (n=30 per group), the data had a non-normal distribution. A comparison between the electroacupuncture and control groups was conducted using the Mann-Whitney U test, which compared the median differences of the outcomes. Differences between groups were calculated by comparing baseline (T1) and endpoint (T10) values.  Page 6 Line 16

 -According to the review comment about the Base line , the revision was made as T1.(Page 6 line 11)

 - All abbreviations in the table were explained at the bottom of all tables. (table 1 and table 3 )

 - According to Sham setting : The authors hypothesized that the reason many measures of cognitive function did not differ between the two groups after 10 weeks of treatment was due to two factors: firstly, the baseline subjective cognitive function in the control group were less severe than those in the experimental group; and secondly, the ceiling effect, as the baseline scores of executive function, subjective cognitive complaints, and quality of life were relatively high in both groups. These points have been addressed in the discussion section.

# Reviewer report: Reviewer response for version 1

**DOI:** 10.5256/f1000research.161025.r282194

**Published:** 2024-06-08

**Authors:** Rosa N Schnyer

**Affiliations:** 1 School of Nursing, The University of Texas, Austin, Texas, USA

The authors set out to conduct a rigorous, double-blind RCT on the effect electro-acupuncture on cognitive symptoms in major depressive disorder. The subject is highly relevant clinically and the study is timely, as it is critical to identify strategies to manage cognitive and mood symptoms which affect a large segment of the population worldwide.

 There are some key issues that need to be addressed in the reporting of the results, some critical considerations in future proposals on this subject:

Protocols: Please refer to and incorporate the STRICTA guidelines and include the checklist https://stricta.info/  STRICTA Standards for Reporting Clinical Trials of Acupuncture, which is an official extension of the CONSORT criteria. Description of the interventions, training of the acupuncture providers, etc. is incomplete. For example, there is no description of the needling parameters or the intensity / frequency of the electrostimulation

Double-Blind: You state that the EG group received EA at 13 points: 10 scalp and 3 body points-weekly 10 weeks while the CG Control, received Brief needling at LI 4 Hegu for 10 weeks. You state that acupuncturists were also blinded, but given the design of the study, it is unlikely that acupuncturists were indeed blinding, when considering the electro protocol vs the sham protocol. Trained acupuncturists would know the difference in point selection, and it’s unlikely that they would not question shallow needling at Hegu as an effective treatment for cognitive symptoms and / or depression.

 You also didn’t assess after the study. whether the participants were in fact blinded to treatment, or whether the blinding work for either participants or providers.

 In the future consider exploring other well proven control for EA, for example by not attempting to blind the acupuncturist and using in the control group a small selection of non-acupuncture points connected to EA for which the wires have been cut but tapped to appear as if the machines work the same as the EG group.

Population description and use of medication (Table 2): One major concern with this study, is the inclusion of patients who are on a variety of psychotropic medications that have effects beyond antidepressant treatment. There doesn’t seem to be explicit consideration for the way in which some of these medications may impact cognitive deficits, and thus confound treatment effects.

 Although the idea of using a naturalistic sample that reflects the complex and varied population experiencing comorbid cognitive symptoms and depression, who are on multiple and different medication regimes is closer to the real world practice in clinical setting, the sample is too small to identify a differential response to treatment, which may be confounded by different medication combinations. This is a limitation of the study that needs to be explicitly included in the discussion section.

 In the future, if conducting a naturalistic trial, you may need a much larger number of patients with these characteristics to detect conclusive differences between groups. Or in a smaller study, limit acupuncture as an augmentation of one specific class of medications only.

Conclusions and Discussion: Your conclusions don’t reflect the results of the analysis; the scores for the CG overall are lower at week 1 and therefore it is possible that this cohort had less severe cognitive decline. Nevertheless, the difference of drop on score of 11.8 in EG vs 18.5 in CG (6.7 seconds) between week 1 and 10 doesn’t seem to be a significant enough difference between EG and CG.

 Therefore it is hard to conclude in the discussion section that “Findings from the study indicate that electrical stimulation acupuncture can significantly reduce subjective cognitive complaints in individuals with mild to moderate depressive symptoms and cognitive issues, when compared to the control group”.  The reduction is not significant when compared to CG.

Other minor:

 Provide consistency when describing the trial: it has been conducted, and is not waiting to be conducted (use of pats vs future verb tense)

Is the work clearly and accurately presented and does it cite the current literature?

Partly

If applicable, is the statistical analysis and its interpretation appropriate?

I cannot comment. A qualified statistician is required.

Are all the source data underlying the results available to ensure full reproducibility?

No source data required

Is the study design appropriate and is the work technically sound?

Partly

Are the conclusions drawn adequately supported by the results?

No

Are sufficient details of methods and analysis provided to allow replication by others?

Partly

Reviewer Expertise:

Acupuncture, depression, pain, women's health

I confirm that I have read this submission and believe that I have an appropriate level of expertise to confirm that it is of an acceptable scientific standard, however I have significant reservations, as outlined above.

# Response

**Published:** 2024-06-26

**Authors:** Muthita Phanasathit

**Affiliations:** Department of Psychiatry, Thammasat University, Pathum Thani, Thailand

**Protocols: Please refer to and incorporate the STRICTA guidelines and include the checklist https://stricta.info/  STRICTA Standards for Reporting Clinical Trials of Acupuncture, which is an official extension of the CONSORT criteria. Description of the interventions, training of the acupuncture providers, etc. is incomplete. For example, there is no description of the needling parameters or the intensity / frequency of the electrostimulation**

 - According to the review comment, the STRICTA 2010 checklist was done and attached in the “Data availability” section.

**Double-Blind: You state that the EG group received EA at 13 points: 10 scalp and 3 body points-weekly 10 weeks while the CG Control, received Brief needling at LI 4 Hegu for 10 weeks. You state that acupuncturists were also blinded, but given the design of the study, it is unlikely that acupuncturists were indeed blinding, when considering the electro protocol vs the sham protocol. Trained acupuncturists would know the difference in point selection, and it’s unlikely that they would not question shallow needling at Hegu as an effective treatment for cognitive symptoms and / or depression.**

**You also didn’t assess after the study. whether the participants were in fact blinded to treatment, or whether the blinding work for either participants or providers.**

**In the future consider exploring other well proven control for EA, for example by not attempting to blind the acupuncturist and using in the control group a small selection of non-acupuncture points connected to EA for which the wires have been cut but tapped to appear as if the machines work the same as the EG group.**

 -According to the review comment about double blind  the revision was made as follows;

 This study was double-blinded RCT. All participants and assessors were unaware of group assignments. at page 5 Line 4

 -Thank you for your advice about blinding assessment after the intervention: We agree that assessing the blinding of all participants and providers is beneficial after the experimental phase to reduce bias. However, the authors will incorporate this into future studies for improvement.

 -Thank you for your advice. about the sham acupuncture  The authors believe that sham acupuncture lacks efficacy and, due to safety concerns, aim to minimize injury and unnecessary infection risks from acupuncture needle insertion at non-acupuncture points connected to EA through wires.

**Population description and use of medication (Table 2): One major concern with this study, is the inclusion of patients who are on a variety of psychotropic medications that have effects beyond antidepressant treatment. There doesn’t seem to be explicit consideration for the way in which some of these medications may impact cognitive deficits, and thus confound treatment effects.**

**Although the idea of using a naturalistic sample that reflects the complex and varied population experiencing comorbid cognitive symptoms and depression, who are on multiple and different medication regimes is closer to the real world practice in clinical setting, the sample is too small to identify a differential response to treatment, which may be confounded by different medication combinations. This is a limitation of the study that needs to be explicitly included in the discussion section.**

**In the future, if conducting a naturalistic trial, you may need a much larger number of patients with these characteristics to detect conclusive differences between groups. Or in a smaller study, limit acupuncture as an augmentation of one specific class of medications only.**

 - According to the review comment, the authors are also concerned about small sample size and confounding factors such as the potential impact of various medications on cognitive deficits. We have included this issue in the study limitations as follows: (page 16 Line 28-41)

 Furthermore, findings from the present study indicate that electroacupuncture might be able to alleviate subjective cognitive complaints in individuals with mild to moderate depressive symptoms and cognitive issues, compared to the control group. However, the clinical significance of the outcomes of electroacupuncture remains unclear.

 The authors did not include a healthy control group, focusing instead on patients with MDD who had subjective complaints and were receiving standard pharmacological treatment. The present study aimed to see how these patients responded to adjunctive electroacupuncture. Patients with MDD often receive various psychotropic drugs, such as benzodiazepines and antipsychotics, which can affect cognitive function and serve as confounding factors. Previous research suggested that acupuncture can influence cognitive function, so the study focused on its use as an adjunctive treatment in real-world settings.

 Future studies may include a much larger number of patients with these characteristics to detect conclusive differences between groups, or in a smaller study, limit acupuncture as an augmentation of one specific class of medications

**Conclusions and Discussion: Your conclusions don’t reflect the results of the analysis; the scores for the CG overall are lower at week 1 and therefore it is possible that this cohort had less severe cognitive decline. Nevertheless, the difference of drop on score of 11.8 in EG vs 18.5 in CG (6.7 seconds) between week 1 and 10 doesn’t seem to be a significant enough difference between EG and CG.**

**Therefore it is hard to conclude in the discussion section that “Findings from the study indicate that electrical stimulation acupuncture can significantly reduce subjective cognitive complaints in individuals with mild to moderate depressive symptoms and cognitive issues, when compared to the control group”.  The reduction is not significant when compared to CG.**

 - According to the review comment, the authors are aware of the interpretation of results, which has limitations in generalizability from statistical significance to clinical significance. Therefore, the authors have addressed this issue in the discussion and limitations sections as follow: (page 14 Line 13)

 Findings from the study indicate that electroacupuncture can statically significantly reduce subjective cognitive complaints in individuals with mild to moderate depressive symptoms and cognitive issues, when compared to the control group.

 -The revision was made by employing past tense.

 Thank you
